# Antiplatelet Usage Impacts Clot Density in Acute Anterior Circulation Ischemic Stroke

**DOI:** 10.3390/ijms17091382

**Published:** 2016-08-23

**Authors:** Slaven Pikija, Jozef Magdic, Anita Lukic, Catharina Schreiber, Johannes Sebastian Mutzenbach, Mark R. McCoy, Johann Sellner

**Affiliations:** 1 Department of Neurology, Christian Doppler Medical Center, Paracelsus Medical University, Salzburg 5020, Austria; s.pikija@salk.at (S.P.); j.mutzenbach@salk.at (J.S.M.); 2 Department of Neurology, Univerzitetni Klinični Center, Maribor 2000, Slovenia; jozef_magdic@yahoo.com; 3 Department of Anesthesiology, General Hospital Varazdin, Varazdin 42000, Croatia; lukic.anita@yahoo.com; 4 Department of Cardiac Surgery, Salzburger Landeskliniken, Paracelsus Medical University, Salzburg 5020, Austria; c.schreiber@salk.at; 5 Division of Neuroradiology, Christian Doppler Medical Center, Paracelsus Medical University, Salzburg 5020, Austria; ma.mccoy@salk.at; 6 Department of Neurology, Klinikum rechts der Isar, Technische Universität München, München 81675, Germany

**Keywords:** ischemic stroke, hyperdense artery sign, antiplatelets, computed tomography, clot

## Abstract

We explored whether clot density in middle cerebral artery (MCA) occlusion is related to clinical variables, stroke etiology, blood constituents, and prestroke medication. We performed a retrospective chart review of patients with acute ischemic stroke of the anterior circulation admitted to two Central European stroke centers. The acquisition of non-contrast enhanced CT (NECT) and CT angiography (CTA) within 4.5 h of symptom onset was obligatory. We assessed the site of MCA occlusion as well as density, area, and length of the clot in 150 patients. The Hounsfield unit values for the clot were divided with contralateral MCA segment to yield relative Hounsfield Unit ratio (rHU). The site of the vessel occlusion (M1 vs. M2) and antiplatelet usage, but not stroke etiology, significantly influenced rHU. We found an inverse correlation of rHU with erythrocyte count (*p* < 0.001). The multivariate analysis revealed that a higher rHU (i.e., clot being more hyperdense) was more likely with the use of antiplatelets (OR 4.24, CI 1.10–16.31, *p* = 0.036). Erythrocyte (OR 0.18, CI 0.05–0.55, *p* = 0.003), and thrombocyte counts (OR 0.99, CI 0.98–0.99, *p* = 0.029) were associated with odds for more hypodense clots (lower rHU). Our study disclosed that antiplatelet therapy impacts the composition of intracranial clots of the anterior circulation.

## 1. Introduction

Aspirin alters clot structure in vitro, resulting in the formation of clots with thicker fibbers and bigger pores [1,2]. This mechanism of action may allow better entanglement of erythrocytes and raise the efficacy of thrombolysis [3,4]. Moreover, aspirin may interfere with inflammatory and immunological processes that contribute to thrombus formation, and importantly the white blood cell fraction within the thrombus [5,6]. The immunohistological work-up of thrombi revealed that the number of CD4+ T cells and CD68+ monocytes was increased in erythrocytic red clots compared with the other morphologic groups [7].

Large vessel occlusion accounts for nearly half of acute ischemic strokes and is associated with 4.5-fold increased odds of death compared to patients with a normal CT angiogram (CT-A) [8]. Factors including location and the presence of collaterals, age and origin of the clot, and thrombus size have been implicated in the success of intravenous thrombolysis with recombinant tissue-plasminogen activator (t-PA). Thrombus composition, a mixture of fibrin/platelet accumulations, red and white blood cells (RBCs and WBCs, respectively), may also play a key role for stroke severity and the efficacy of recanalization efforts [9,10].

In acute ischemic stroke, the hyperdense artery sign (HAS) on non-contrast enhanced computed tomography (NECT) is a surrogate of intraluminal thrombus [1]. Thrombi with lower clot density, as assessed quantitatively by Hounsfield Units (HU) on NECT, are more resistant to pharmacological lysis and mechanical thrombectomy [10]. Cardioembolic thrombi forming in regions of stasis or low flow are mainly composed of entrapped RBCs. In contrast, fibrin and platelets are the main components of atherosclerotic thrombi. There is emerging evidence that clots undergo further modification of their composition after occluding an artery [11,12,13]. Yet, variables which may impact this process in the setting of the occlusion of intracranial arteries have not been evaluated so far.

Here, we tested whether prior antiplatelet usage is associated with in vivo clot composition in patients with acute anterior circulation ischemic stroke.

## 2. Results

We evaluated a total of 4629 CT scans, and a HAS was detected in 230. The 150 patients with CT-A-confirmed occlusion in the M1 or M2 segments underwent further analysis. Details of demographics, clinical variables, stroke etiology, radiological findings, and treatment are reported in Table 1. Standard aspirin dosage was 100 mg per day.

### 2.1. Normal-Appearing Contralateral Artery (CA)

We found an association between erythrocyte count and density of the CA (*p* = 0.047, *rho* = 0.324). Fibrinogen levels were not associated with the CA density (*p* = 0.199, *rho* = −0.256).

### 2.2. Clot Density

Fibrinogen levels were negatively correlated with clot density (*p* = 0.046, *rho* = −0.165).

### 2.3. Relative Hounsfield Unit Ratio (rHU) (Clot Density/CA Density)

**Figure 1:**
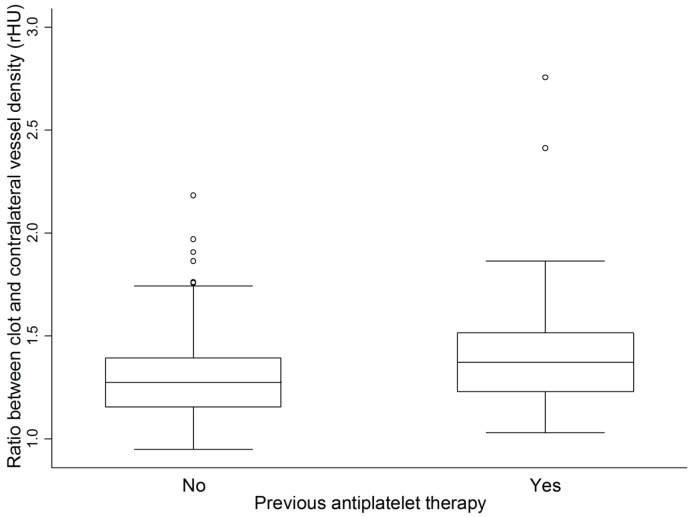
Analysis of relative clot density (rHU) in 150 patients with and without intake of antiplatelets (*p* = 0.024).

The rHU was significantly lower for a HAS located in the M1 segment compared to a distal occlusion (*p* = 0.019). rHU was higher in patients on antiplatelets prior to stroke (*p* = 0.024, Figure 1). In contrast, this could not be shown for the patients receiving anticoagulants (*n* = 18). This finding was pronounced in the analysis of M2 occlusions (*p* = 0.021), whereas no impact of prior antiplatelet usage was found in thrombi occluding the M1 segment (*p* = 0.419). The erythrocyte count was negatively correlated with rHU (*p* < 0.001, *rho* = −0.324). We did not identify an association of fibrinogen levels with rHU. The thrombocyte count was negatively related to rHU in the M2 segment only (*p* = 0.05, *rho* = −0.626). We did not find differences of rHU by TOAST etiology, or by cardioembolic vs. other etiologies. Area and length of the thrombus did not correlate with the rHU. This finding was corroborated in patients with cardioembolic vs. other etiologies. In a subgroup of patients that underwent mechanical thrombectomy, there was no correlation with length, area, or history of antiplatelets with recanalization.

### 2.4. Clinico-Radiological Outcome

**Figure 2:**
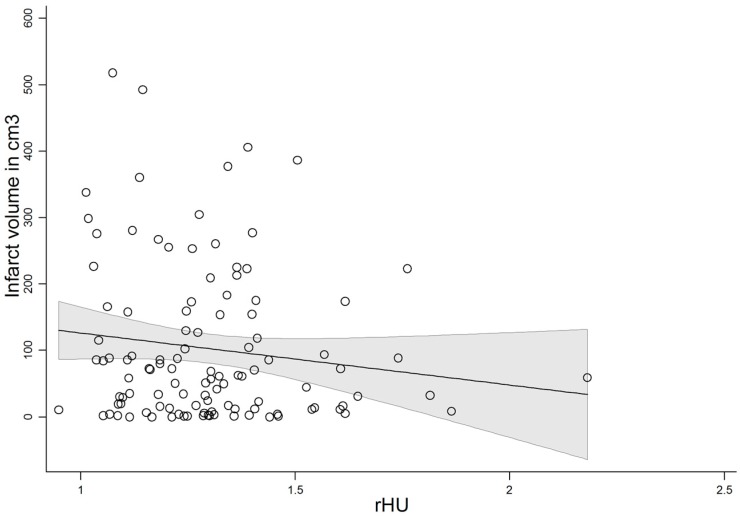
Correlation of final infarct volume on non-contrast enhanced computed tomography (NECT) with relative clot density (rHU) in 133 stroke patients presenting within 4.5 h after symptom onset (*p* = 0.048). rHu—ratio between clot and contralateral vessel density.

A history of antiplatelet usage or rHU was not predictive of clinical outcome. There was a trend for lower rHU with greater final infarct volume (*n* = 139, *p* = 0.068). The correlation was significant when the patients with complete resolution were excluded (*p* = 0.048, *n* = 133, Figure 2).

### 2.5. Multivariate Analysis

Stepwise logistic regression analysis incorporated potential confounders. We found that atrial fibrillation, erythrocyte and thrombocyte count, history of antiplatelet usage, and MCA segment were independent predictors for rHU (Table 2).

## 3. Discussion

Our study disclosed an effect of antiplatelet therapy on the composition of intracranial clots in the setting of acute ischemic stroke in the anterior circulation. The relative clot density is significantly higher with the history of antiplatelet usage but lower with higher erythrocyte and thrombocyte count.

Traditionally, it was thought that the composition (and hence the density) of MCA thrombi was primarily related to the embolic source [2]. In this concept, thrombi embolized from low-flow sources such as the left atrial appendage are more hyperdense by the substantial composition made up by erythrocytes. In contrast, thrombi from high-flow sources such as the carotid artery with a higher proportion of fibrinogen are supposed to be less attenuating. In this regard, a recent histological study of clot material extracted during mechanical recanalization and further reports challenged this assumption [11]. Moreover, clot material might undergo in situ transformation after occluding a vessel. While only invasive retrieval of embolized clot material could provide exact histological analysis, some characteristics of clot material can be evaluated by the amount of X-ray absorption of the clot (i.e., its density) [1].

Aspirin, as taken by the majority of patients on antiplatelets in our study, has been shown to lead to better clinical outcome after acute ischemic stroke [14]. The background of these beneficial effects are likely to be multifaceted. Potential mechanisms of action may be the mitigation of consequences of acute stroke through compensatory vasodilatation, prevention of free radical-induced injury, or limitation of further thrombus extension and alteration of its composition [14]. Finally, the chances for successful thrombolytic intervention increase with clot density [9].

The presence of collaterals and the location of the vessel occlusion may impact local changes in clot composition [15]. Distal intracerebral vessels such as the M2 segment may be more isolated from the surrounding blood than their proximal counterparts. We hypothesized that the in situ modification of clot composition may be noticed by changes of plasma constituents involved in blood coagulation. Remarkably, we found a negative association of clot density with fibrinogen concentration. This confirms the hypothesis that a clot “loses” its density by potential acquisition of fibrin from the surrounding blood. Since the erythrocyte count showed no association with the clot density, it is reasonable to assume that they did not constitute part of additional clot material being formed in situ. In contrary, the density of the non-affected vessel showed a direct association with the erythrocyte count. Contrary to previous reports, we could not confirm a correlation of radiological clot properties with the recanalization or previous usage of antiplatelets with favorable clinical outcome [10,14]. However, the borderline association of lower rHU and more extensive final infarct volume found in this study is in line with the literature [10].

A recent study of mechanically-extracted MCA thrombi reported that HAS reflects the early phase of thrombus composition [16]. Thus, further studies need to focus on the time course of thrombus modification. Prospective studies may also take potentially clot-modifying factors such as smoking and laboratory markers of platelet endothelial activation into account [17]. Moreover, the patients could have been resistant to antiplatelets or may have been incompliant for regular intake, and we did not employ functional analysis or interview to rule out this bias.

The shortcoming of our study is the retrospective design, which is inherently prone to various biases, the limited size of the cohort, and the relatively low number of patients on antiplatelets. Additionally, we did not include patients without CTA imaging. Future studies should also consider measures to confirm compliance for the intake of antiplatelets. Moreover, differences in demographics and medical history between the patients on and not on antiplatelets need to be taken into account. These include age, prior history of TIA/stroke, and presence of arterial hypertension.

## 4. Material and Methods

### 4.1. Study Design

We performed a retrospective study at two Central European tertiary care stroke centers: Christian Doppler Medical Center, Salzburg, Austria (CD) and University Medical Centre Maribor, Maribor, Slovenia (MB). Study periods at CD were June 2012—December 2015 and at MB January 2011—January 2015. We collected data exclusively by retrospective chart review. Subsequent analyses were performed on anonymized data, and therefore ethical approval was not required according to national regulations in both countries. Experienced stroke physicians (SP and JM) blinded to the clinical and treatment data and any subsequent imaging did the CT scoring and reached consensus in the case of discrepancy.

### 4.2. Patient Selection

We screened all patients admitted for acute ischemic stroke of the anterior circulation with a NECT and CT-A within 4.5 h of symptom onset. We excluded patients with additional pathologies such as brain tumors, arteriovenous malformations, and traumatic brain injury, and with CT of limited quality. We recorded symptom-to-CT and time-to-control CT time points. The CT scanner at CD was a Sensation 64 (Siemens, Erlangen, Germany) and in MB an Aquilion 64 (Toshiba Medical Systems, Otawara, Japan). NECT scans were reconstructed into 4 mm-thick adjacent slices through the whole brain. The treatment and stroke unit care adhered to national guidelines in both centers. Minimal diagnostic work-up included laboratory examinations, Doppler sonography of the carotid arteries, transthoracal echocardiography, and 24 h ECG monitoring.

### 4.3. Quantification of the Clot Density

We recorded the presence, location, and extent of HAS on NECT. The definition of HAS was derived from an earlier publication as the presence of a more hyper-attenuating intracranial artery compared to adjacent or equivalent contralateral vessels [18].

We used the anatomical classification for middle cerebral artery (MCA) branches proposed by Goyal et al.: M1 was defined as the portion of the MCA to the first major bifurcation in the Sylvian fissure, and the M2 segment as the subsequent part in the fissure [19]. We manually delineated the hyperdense area for the determination of length and density of the thrombus by using the IMPAX software. Regions that included vessel wall calcification (>90 HU) were excluded from the region of interest (ROI). For determination of the HU sum, the average HU was obtained from all voxels within the ROI and summed across all slices (if present on more than one) [10]. The final HU value was calculated by dividing HU sum by number of slices. We manually circumscribed the hyperdense vessel to record the area (in mm^2^) and length (in mm) of the clot. Both values were summed across slices if present in more than one. The length was measured only when judged to be meaningful—e.g., it was not measured when an isolated “dot sign” in M2 section of the MCA was seen.

In order to correct for hematocrit values, we measured the density of the vessel contralateral to the affected one, as described previously [10]. In this regard, we calculated the relative HU ratio (rHU), which is the final HU value divided by average HU of contralateral non-affected artery. Neurological deficits were assessed by National Institutes of Health Stroke Scale (NIHSS) score.

### 4.4. Quantification of the Final Infarct Volume and Recanalization

The follow-up CT scans, performed more than 24 h and less than 7 days from symptom onset, were examined for the presence and size of infarction. The hypodense area was delineated manually on each CT slice (4 mm), which yielded the area in cm^2^. The volume in cm^3^ was derived from the measured area and the corresponding slice thickness. Thrombolysis in cerebral intervention score (TICI) denoted the extent of reperfusion after neurointervention and is defined as follows: 0—no reperfusion; 1—penetration, no distal filling; 2a—perfusion, <50% distal filling; 2b—perfusion, >50% distal filling; 3—full perfusion.

### 4.5. Data Analysis

Patients’ demographics, medical history, imaging features and clinical outcome were summarized using descriptive statistics. Categorical variables were compared using Fisher’s exact or χ^2^ test, while continuous variables were compared using the Mann–Whitney test for independent samples. The association between the variables was assessed by rank correlation. The relationship between different variables was analyzed using stepwise multiple regression. All statistical analyses were performed with MedCalc (MedCalc Software, Mariakerke, Belgium).

## 5. Conclusions

In conclusion, our findings point at a potential effect of antiplatelet usage upon clot composition within the intracranial artery. This could explain previously observed favorable effects of antiplatelets on stroke severity and varying success of recanalization efforts [20,21].

## Figures and Tables

**Table 1:** Characteristics of the study cohort from two stroke centers (*n* = 150).

Characteristics (N)	Antiplatelet Treatment ^†^	Without Antiplatelet Treatment	*p* Value
Age (years) *	80 (70, 86; *n* = 40)	72 (57, 82; *n* = 110)	0.003
Men	20 (28.6%)	50 (71.4%)	0.622
**Medical History**			
Prior stroke/TIA (*n* = 21)	10 (47.6%)	11 (52.4%)	0.019
Atrial fibrillation (*n* = 71)	23 (32.4%)	48 (67.6%)	0.133
Peripheral artery disease (*n* = 8)	3 (37.5%)	5 (62.5%)	0.476
Carotid stenosis >50% (*n* = 24)	9 (37.5%)	15 (62.5%)	0.190
Arterial hypertension (*n* = 103)	35 (33.9%)	68 (66.0%)	0.003
Diabetes mellitus (*n* = 21)	5 (23.4%)	16 (76.2%)	0.750
Chronic heart failure (*n* = 24)	9 (37.5%)	15 (62.5%)	0.190
Use of anticoagulants (*n* = 18)	0	18 (100.0%)	0.006
**Stroke Type by TOAST**			
Cardioembolic (*n* = 73)	23 (31.5%)	50 (68.5%)	0.343
Unknown (*n* = 42)	7 (16.7%)	35 (83.3%)	
Large artery atherosclerosis (*n* = 26)	8 (30.8%)	18 (69.2%)	
Other (*n* = 9)	2 (22.2%)	7 (77.8%)	
**Clinical Presentation**			
NIHSS (150) *	18.5 (13.5, 22)	17.0 (11.0, 20.0)	0.054
Serum glucose (mg/dL) (*n* = 148) *	120.0 (109.0, 144.3)	117.0 (103.7, 136.0)	0.239
Erythrocytes (×10^12^/L) (*n* = 117) *	4.4 (3.6, 4.8)	4.5 (4.2, 4.7)	0.142
Thrombocytes (×10^9^/L) (*n* = 117) *	219 (170, 280)	197 (238, 275)	0.322
Hematocrit (%) (*n* = 117) *	39.5 (33.6, 43.0)	40.5 (37.4, 42.6)	0.273
HbA1c (mmol/L) (*n* = 101) *	5.4 (5.1, 5.8)	5.6 (5.4, 5.8)	0.1258
Fibrinogen (mg/dL) (*n* = 146) *	356 (304, 473)	348 (305, 425)	0.613
C-reactive protein (mg/L) (*n* = 101) *	0.3 (0.2, 1.3)	0.35 (0.18, 0.9)	0.648
**Acute Treatment**			
Thrombolysis (t-PA, *n* = 123)	33 (26.8%)	90 (73.2%)	0.923
Thrombectomy (*n* = 87)	21 (24.1%)	66 (75.9%)	0.411
Thrombolysis + Thrombectomy (*n* = 75)	17 (22.7%)	58 (77.3%)	0.268
**Thrombectomy Outcome [TICI]** **(*n* = 83)**			
No perfusion (0) (*n* = 16)	5 (31.2%)	11 (68.7%)	0.590
Penetration, no distal filling [1] (*n* = 2)	0 (0%)	2 (100.0%)	
Perfusion, <50% distal filling [2a] (*n* = 4)	2 (50.0%)	2 (50.0%)	
Perfusion, >50% distal filling [2b] (*n* = 19)	4 (21.0%)	15 (78.9%)	
Full perfusion [3] (*n* = 42)	9 (21.4%)	33 (78.6%)	
Inadequate (0–2a total) (*n* = 22)	7 (31.8%)	15 (68.2%)	0.323
Adequate (2b–3 total) (*n* = 61)	13 (21.3%)	48 (78.7%)	
**Imaging Characteristics**			
Symptoms to image (min) (*n* = 150) *	95.5 (73.5. 116.5)	87.5 (66.5, 128)	0.619
Affected vessel (*n* = 150)			
Middle cerebral artery M1 (*n* = 120)	30 (25.0%)	90 (75.0%)	0.356
Middle cerebral artery M2 (*n* = 30)	10 (33.3%)	20 (66.7)	
Clot area (mm^2^) (*n* = 147) *	23.8 (13.0, 40.3)	27.1 (14.5, 47.0)	0.499
Clot-relative Hounsfield Unit ratio (rHU) (*n* = 150)	1.37 (1.22, 1.51)	1.27 (1.15, 1.39)	0.024
Final infarct volume (cm^3^) (*n* = 139) *	58.8 (16.2, 149.9)	41.2 (7.2, 138.2)	0.366
Outcome on control imaging (*n* = 141)			
Infarction (*n* = 106)	28 (26.4%)	78 (73.6%)	0.275
Hemorrhagic infarction (*n* = 28)	8 (28.6%)	20 (71.4%)	
Resolution (infarct volume = 0) (*n* = 7)	0	7 (100%)	
**Clinical Outcome**			
In-hospital mortality (30 of 150)	9 (30%)	21 (70%)	0.644
NIHSS at discharge (points) (130) *	11 (4, 18)	6 (2, 13)	0.102

Data are presented as median (25, 75 percentile) or count (percent). HbA1c—glycated hemoglobin; NIHSS—National Institutes of Health Stroke Scale; rt-PA—recombinant human tissue plasminogen activator; TIA—transitory ischemic attack; TICI—thrombolysis in cerebral infarction grading; TOAST—Trial of Org 10172 in Acute Stroke Treatment. † aspirin (100 mg per day), clopidogrel; * number of patients available for analysis.

**Table 2:** Odds of detecting a clot with higher relative Hounsfield density. Results of the multivariate analysis of 116 patients, with the CT in the first 4.5 h after symptom onset.

Independent Predictors for Higher rHU	OR (95% CI)	*p*
Use of Antiplatelets	4.24 (1.10–16.3)	0.036
Erythrocyte Count	0.18 (0.05–0.55)	0.003
Thrombocyte Count	0.99 (0.98–0.99)	0.029
Vessel Affected (M1 vs. M2)	9.03 (1.36–59.73)	0.022
History of Atrial Fibrillation	2.96 (1.07–8.20)	0.036
